# A Bivalent Live Attenuated Influenza Virus Vaccine Protects against Drifted H1N2 and H3N2 Clinical Isolates in Swine

**DOI:** 10.3390/v15010046

**Published:** 2022-12-23

**Authors:** Lauren Aubrey, Ulises Barron-Castillo, Susan Detmer, Yan Zhou

**Affiliations:** 1 Vaccine and Infectious Disease Organization (VIDO), University of Saskatchewan, Saskatoon, SK S7N 5E3, Canada; 2 Vaccinology and Immunotherapeutics Program, School of Public Health, University of Saskatchewan, Saskatoon, SK S7N 2Z4, Canada; 3 Department of Veterinary Microbiology, Western College of Veterinary Medicine, University of Saskatchewan, Saskatoon, SK S7N 5B4, Canada; 4 Department of Veterinary Pathology, Western College of Veterinary Medicine, University of Saskatchewan, Saskatoon, SK S7N 5B4, Canada

**Keywords:** influenza, vaccine, swine

## Abstract

Influenza A viruses (IAVs) can cause a highly contagious respiratory disease for many mammalian species. In pigs, IAVs cause high morbidity and low mortality disease in susceptible populations that can have significant financial and production impacts. They can also present opportunities for mutations and gene reassortment, producing influenza strains with pandemic potential. Therefore, it is very important to prevent and control influenza infection in pigs, and the chief way to do so is through vaccination. The subtypes of IAV most prevalent in swine across the world are H1N1, H1N2, and H3N2; however, genetic diversity of these viruses can vary greatly by region. We previously developed an elastase-dependent bivalent live attenuated vaccine using two Canadian swine influenza A virus (swIAV) isolates, A/Swine/Alberta/SD0191/2016 (H1N2) [SD191] and A/Swine/Saskatchewan/SD0069/2015 (H3N2) [SD69], which provided protection against homologous strains. In this study, we demonstrate that this vaccine extends protection in pigs to more current, drifted non-homologous H1N2 and H3N2 strains, A/Swine/MB/SD0467/2019 (H1N2) [SD467] and A/Swine/AB/SD0435/2019 (H3N2) [SD435]. The vaccine elicited a robust immune response in the serum and the lung and reduced viral replication as well as lung pathology associated with these strains. Therefore, this bivalent vaccine remains a strong candidate that would be beneficial to the swine influenza vaccine market in North America.

## 1. Introduction

Influenza A viruses (IAV) are a significant pathogen for many species, including swine. Infection can cause highly contagious respiratory disease in pigs [1]. Influenza infection in swine leads to mild disease with very low mortality, but morbidity rates in a herd can reach 100% [2]. This results in economic losses for farmers due to decreased weight gain in infected pigs, decreased production performance, and reproductive failure in sows [3,4]. Co-infection of influenza with other swine respiratory pathogens can also lead to the development of porcine respiratory disease complex, and in consequence, increased mortality and economic losses [5].

Additionally, pigs are susceptible to infection with avian and human IAVs as well as swine IAV (swIAV) due to the presence of both avian and mammalian sialic acid galactose linkages in their respiratory tract [6]. This makes it possible for reassortment to occur when co-infected with multiple strains, which could lead to the production of new strains with pandemic potential [4,7]. As humans have similar sialic acid galactose linkage distribution throughout the respiratory tract, bi-directional spillover events are possible between human and swine. The first known occurrence of this was during the 1918 influenza pandemic, when IAV was introduced to swine from humans [8]. This lineage remained stable in porcine populations until the 1990s, when human and avian H3N2 strains reassorted with the circulating H1N1 lineage to produce double and triple reassortant strains of H1N1, H1N2, and H3N2 subtypes [8,9]. The newly developed triple reassortant gene (TRIG) cassette led to a period of rapid IAV diversification in North American swine [10]. This TRIG cassette is very stable and facilitates the substitution of different HA and NA combinations [5]. Since 2005, many strains with this internal TRIG cassette paired with human HA and NA genes spread across swine herds in the USA [9]. The next notable spillover event occurred in 2009, when a swine-origin H1N1 strain (H1N1pdm2009) spread to humans, leading to the 2009 influenza pandemic [11]. Human-to-swine transmission (reverse zoonosis) of the human pandemic H1N1 IAV (H1pdm) was recorded multiple times in North American swine, which led to the establishment of a new lineage and new reassortant swIAVs [8,10,12]. In total, seven antigenically distinct H1 clades and four distinct clades of H3 viruses have been recorded in North American swine [5]. Recently, an swIAV surveillance program in Asia identified a predominant Eurasian avian-like (EA) reassortant genotype 4 (G4) virus, with H1pdm and triple reassortant internal genes. This G4 virus was measured in 10.4% of swine workers tested, and presents markers of pandemic potential, with the ability to transmit to humans and different antigenicity from currently circulating human viruses [7]. Therefore it is very important to prevent and control influenza infection in pigs from both a swine industry and public health perspective, and the chief way to do so is through vaccination [4].

In the USA, the most common type of vaccine is whole inactivated virus (WIV), but an RNA vector vaccine expressing HA and an NS1-truncated live attenuated influenza virus vaccine (LAIV) have also been approved [4]. The subtypes of IAV most prevalent in swine across the world, including North America, are H1N1, H1N2, and H3N2 [13]. Genetic diversity of these viruses can vary greatly by region, however, and there are differences in the genetic evolution of swIAV in Canada and the USA, particularly in H1 subtype viruses [12]. Surveillance in Canada between 2009 and 2016 indicated that the genetic clades of H1 that were dominant in the USA, H1g (1A.3.3.3) and H1d-1 (1B.2.2), were not detected in Canada, and the H1 viruses in Canada were highly divergent from those of the USA [12]. A new H1 clade was identified (H1a-3) in Canada that began in Manitoba and underwent rapid growth before spreading across the country and into the USA. With regards to H3 viruses, six H3 lineages were documented in American swine (IV-A to IV-F), and of those, three were found in Canadian swine (IV-B, IV-C and IV-E) [12]. This highlights the importance of regional surveillance and awareness of circulating strains to inform effective vaccine programs, and indicates that vaccines designed based on IAV circulating in American swine may not provide protection in Canadian swine [12].

Previously, a bivalent vaccine was created using two Canadian swIAV isolates, A/Swine/Alberta/SD0191/2016 (H1N2) [SD191] and A/Swine/Saskatchewan/SD0069/2015 (H3N2), which are representatives for the H1α-3 antigenic group and a new H3 antigenic group from Western Canada (swine cluster IV-E), respectively [14]. This novel vaccine is a live attenuated virus vaccine made using elastase-dependent forms of SD191 and SD69, SD191−R342V, and SD69-K345V. Studies showed that it was protective against both SD191 and SD69, as well as a heterologous H1N2 strain A/Swine/Saskatchewan/SD0142/2015 (H1N2) that was also isolated from Western Canada [14]. Since then, circulating swIAV strains have continued to drift. More recent clinical isolates from swine in Western Canada were collected and isolated from field samples at the Western College of Veterinary Medicine, University of Saskatchewan, Saskatoon, SK, Canada. A/Swine/MB/SD0467/2019 (H1N2) [SD467] is a member of the Hα-3 antigenic group but has five amino acid substitutions out of 54 key H1 antigenic sites as compared to SD191 [10,15,16]. A/Swine/AB/SD0435/2019 (H3N2) [SD435] is a member of the IV-E cluster but has drifted to include two amino acid substitutions out of the six key H3 antigenic sites [17].

In this study, we evaluated whether the bivalent elastase-dependent LAIV would hold up against new clinical isolates, and can report that it provided protection in swine when challenged with currently circulating swIAV strains SD467 (H1N2) and SD435 (H3N2).

## 2. Materials and Methods

### 2.1. Cells and Viruses

Madin-Darby canine kidney (MDCK) (ATCC, #CRL-2936) cells were maintained in Minimal Essential Medium (MEM) (Sigma-Aldrich, M4655, St. Louis, MO, USA) containing 10% fetal bovine serum (FBS) (Thermo Fisher Scientific, Ottawa, ON, Canada 16000-044), and were kept in a humidified 5% CO_2_ incubator at 37 °C. A/Swine/Alberta/SD0435/2019 (H3N2) [SD435] and A/Swine/Manitoba/SD0467/2019 (H1N2) [SD467] swIAV were isolated from field samples at the Western College of Veterinary Medicine, University of Saskatchewan, Saskatoon, SK, Canada. The vaccine viruses SD191−R342V and SD69-K345V were rescued as previously described [14]. All viruses were grown in MDCK cells in the presence of 0.2% bovine serum albumin (BSA) (Sigma-Aldrich, A7030) with either 1 μg/mL L-[(toluene-4-sulphonamido)-2-phenyl] ethyl chloromethyl ketone (TPCK)-trypsin (WT viruses) or 0.5 μg/mL human neutrophil elastase (elastase-dependent viruses) (Sigma-Aldrich, E8140).

### 2.2. Animal Trial Design

**Figure 1:**
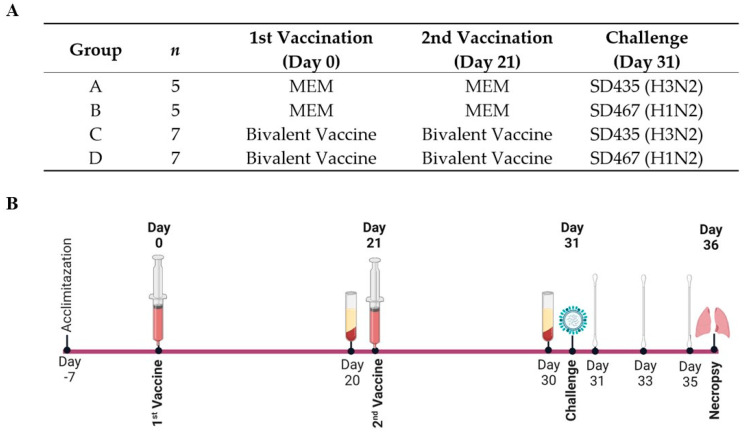
The grouping of pigs and trial design for evaluating the protective efficacy of the bivalent LAIV against new clinical isolates. Pigs (*n* = 5 for MEM/MEM groups and *n* = 7 for bivalent/bivalent groups) were intratracheally vaccinated with 4 mL of MEM or the bivalent vaccine composed of 1 × 10^6^ PFU of each SD191−R342V and SD69-K345V on days 0 and 21. On day 31, the pigs were intratracheally challenged with MEM or 1 × 10^6^ PFU of either SD435 (H3N2) or SD467 (H1N2). (**A**) The schedule of immunization, challenge, and sample taking for this animal trial. Twenty-four four-week-old swIAV-negative pigs were allowed to acclimatize for seven days prior to infection. At five weeks of age (day 0) as well as at eight weeks of age (day 21), the pigs in groups A and B were intratracheally mock vaccinated with 4 mL of MEM, while groups C and D were vaccinated with a bivalent vaccine containing 1 × 10^6^ PFU of each SD191−R342V and SD69-K345V in 4 mL MEM. Ten days later (day 31), the pigs were challenged with either MEM (mock) or 1 × 10^6^ PFU of either SD435-WT (H3N2) or SD467-WT (H1N2). The pigs were monitored for five days post-challenge, with rectal temperatures taken daily and nasal swabs taken from both nostrils on days 1, 3, and 5. Serum was collected after the first (day 20) and second (day 30) vaccinations. On day 5 post-challenge (day 36) all pigs were humanely euthanized, and the lungs were extracted for evaluation. (**B**) Created with BioRender.com.

Twenty-four four-week-old swIAV-negative pigs were obtained from the Prairie Swine Centre Inc. (Saskatoon, SK, Canada). These pigs were randomly selected and divided into four groups with seven pigs per vaccinated group, and five pigs per mock vaccinated group. The group assignment is described in Figure 1A. These groups were housed in separate rooms based on vaccination groups (groups A + B and C + D housed together) and allowed to acclimatize for seven days prior to infection. At five weeks of age (day 0) as well as at eight weeks of age (day 21), the pigs in groups A and B were intratracheally mock vaccinated with 4 mL of MEM, while groups C and D were vaccinated with a bivalent vaccine containing 1 × 10^6^ PFU of each SD191−R342V and SD69-K345V in 4 mL MEM. Ten days later (day 31), the pigs were challenged with either MEM (mock) or 1 × 10^6^ PFU of either SD435-WT (H3N2) or SD467-WT (H1N2). The pigs were monitored for five days post-challenge, with rectal temperatures taken daily and nasal swabs taken from both nostrils on days 1, 3, and 5. Serum was collected after the first (day 20) and second (day 30) vaccinations for the serum virus neutralization (SVN) assay and enzyme-linked immunosorbent assay (ELISA). On day 5 post-challenge, all pigs were humanely euthanized, and the lungs were extracted and evaluated for the presence of swIAV characteristic gross lesions. Lung tissue samples were also collected for virus isolation (Figure 1B).

### 2.3. Ethics Statement

All animal procedures were approved by the University Animal Care Committee (UACC) and Animal Research Ethics Board (AREB) of the University of Saskatchewan. This protocol was approved on 12 November 2021 (Animal Use Protocol #20190064). All procedures were performed in accordance with the standards required by the Canadian Council of Animal Care (CCAC) at the Vaccine and Infectious Disease Organization (VIDO), University of Saskatchewan, Saskatoon, SK, Canada.

### 2.4. Sampling

Nasal swabs from each nostril were placed into 1 mL of MEM containing 1× antibiotic-antimycotic (Thermo Fisher Scientific, Ottawa, ON, Canada, 15240-062) and frozen at −80 °C until qRT-PCR was performed. All pigs were humanely euthanized by intravenous administration of euthanyl (240 mg/mL sodium pentobarbitol; 2 mL per 4.5 kg). Upon euthanasia, the lungs were removed in toto to determine both the percentage of purple-red, firm lesions as well as pneumonia. The percentages were determined based on the lung lobe weights as well as the entire lung volumes [18]. Lung samples were also taken from the right apical, cardiac, and diaphragmatic lobes for viral titration. These lung samples were mixed in equal volumes of 10% *w*/*v* MEM containing 1× antibiotic-antimycotic for titration.

### 2.5. Enzyme-Linked Immunosorbent Assay (ELISA)

To make coating antigen, SD435 and SD467 were propagated in MDCK cells and purified using sucrose gradient ultracentrifugation. Inactivation of the viruses occurred by adding 97% β-propiolactone to the virus at a concentration of 1:1000 (*v*/*v*) (Thermo Fisher Scientific, AAB2319703). This mixture was rocked at 4 °C overnight, incubated at 37 °C for two hours to facilitate hydrolysis of β-propiolactone, then stored at −80 °C until use.

To measure the swIAV-specific IgG levels induced by vaccination and challenge, pig serum was taken after the first (day 20) and second (day 30) vaccinations, and before necropsy (day 36). Purified β-propiolactone-inactivated viruses SD435 (1 μg/mL), and SD467 (2 μg/mL), diluted in carbonate/bicarbonate coating buffer, (pH 9.6) were applied to Immulon-2 96-well plates at 100 μL/well (Thermo Labsystems, Ottawa, ON, Canada, 3655) and incubated overnight at 4 °C. After overnight incubation, the coated plates were washed four times with TBST (0.1 M Tris, 0.17 M NaCl, and 0.05% Tween 20), to which four-fold serial dilutions of the serum or BALF were added to the plate in duplicate, followed by a two-hour incubation at room temperature. Serum was added at a starting dilution of 1:10, and BALF was added undiluted. Samples of previously defined positive control sera and the appropriate negative controls, serum, and BALF from unvaccinated pigs in a previous study were run on each plate [14]. The plates were washed four times with TBST, after which goat anti-swine IgG (H + L) phosphatase-labeled affinity purified antibody (1:5000) (Sigma Aldrich, SAB3700435) or mouse anti-pig IgA (Serotec, MCA658) (1:300) diluted in TBST was added and left to incubate at room temperature for one hour. The IgA ELISAs were developed by the addition of biotinylated goat anti-mouse IgG (H + L) antibodies (CALTAG, Burlingame, CA, USA, M30015) and streptavidin alkaline phosphatase solution (Jackson ImmunoResearch, West Grove, PA) both for one hour at room temperature. Following incubation, both IgG and IgA plates were washed four times with TBST, to which p-nitrophenyl phosphate substrate (PNPP) [10 mg/mL p-nitrophenyl phosphate di(tris) salt crystalline (Sigma-Aldrich), 1% diethanolamine (Sigma-Aldrich), 0.5 mg/mL MgCl2, and pH 9.8] (1 mg/mL) was added and incubated at room temperature for two hours. The reaction was halted through the addition of 0.3 M ethylenediaminetetraacetic acid (EDTA), and the plates read in a spectrophotometer at 405 nm with a reference of 490 nm. The titer of sample was defined as the highest dilution at which the OD of that sample was higher than the defined cutoff (the mean OD of a known negative sample plus two times the standard deviation).

### 2.6. Virus Neutralization (VN) Assay

MDCK cells (3.5 × 10^4^) were plated into 96-well plates. Serum and BALF were heat inactivated at 56 °C for 30 min. Two-fold dilutions of the serum and BALF were added to the plate in quadruplicate, and 60 µL of diluted serum or BALF was incubated with equal volume of SD435 or SD456 containing 100 TCID_50_ at 37 °C for 1 hour. 100 µL of the mixture was then added to the MDCK cells, and the cytopathogenic effect (CPE) was documented at 48 hours and 72 hours post-infection (hpi). The neutralization antibody titer was the highest dilution of each serum sample that completely protected the cells from CPE in at least 2 out of 4 wells.

### 2.7. Viral Determination

Upon collection, the lung samples were immediately placed on ice and frozen at −80 °C until processing. For processing, each lung tissue was weighed and a 10% (*w*/*v*) concentration of MEM supplemented with 1× antibiotic-antimycotic (Thermo Fisher Scientific, 15240-062) was added. Lung tissue was homogenized in the TissueLyser II (Qiagen, Hilden, Germany) at 30 Hz for 5 min, followed by centrifugation at 5000× *g* for 10 min at 4 °C. The homogenized supernatant was collected and stored at −80 °C until further analysis. The nasal swabs were vortexed for 15 seconds and centrifuged at 1600× *g* for 25 min at 4 °C. The supernatants were collected and stored at −80 °C until further analysis. The viral titers were determined by TCID_50_ assay for the lung and quantitative RT-PCR for the nasal swabs.

### 2.8. RNA Extraction and Quantitative RT-PCR (qRT-PCR)

To determine viral RNA levels of SD467 and SD435 in the nasal swabs post-challenge, qRT-PCR was performed. A standard curve was made using RNA extracted from SD435 and SD467 of a known titer. Briefly, the RNeasy Plus Mini Kit (Qiagen, Toronto, ON, Canada, 74136) was used to extract vRNA from 200 µL of nasal wash. vRNA was converted to cDNA using the universal influenza primer Uni12 and SuperScript III Transcriptase (Invitrogen, Burlington, ON, Canada) [19]. qPCR was performed in triplicate on a StepOnePlusTM Real-Time PCR system (Applied Biosystems, CA, USA) with the Power SYBR Green PCR Master Mix (Applied Biosystems), 5 µL cDNA, and 1 µL of 10µM forward and reverse primers. PCR reactions were run at an annealing temperature of 58 °C for 40 cycles. All sequences of qPCR primers are available upon request.

### 2.9. Statistical Analysis

Statistical analysis was performed using GraphPad Prism 8 software. The Mann-Whitney and Kruskal-Wallis non-parametric tests were used. Significant differences are denoted by * (*p* < 0.05), ** (*p* < 0.01), *** (*p* < 0.001), or **** (*p* < 0.0001). ns = not significant.

## 3. Results

### 3.1. Vaccination with the Bivalent Vaccine Provided Protection against New Clinical Isolates

**Figure 2:**
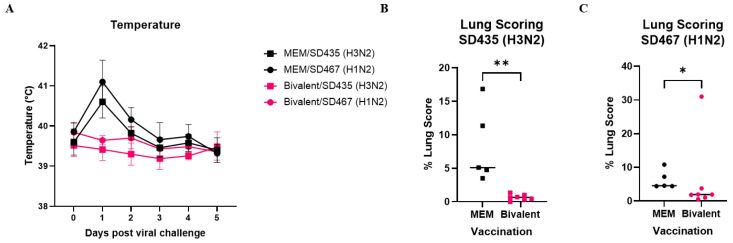
The rectal temperatures and macroscopic lung lesions of pigs vaccinated with the bivalent vaccine followed by swIAV challenge. Pigs were vaccinated twice with a bivalent vaccine composed of SD191−R342V (1 × 10^6^ PFU) and SD69-K345V (1 × 10^6^ PFU) on days 0 and 21. On day 31, the pigs were intratracheally challenged with 1 × 10^6^ PFU of either SD435 or SD467 and then monitored daily for 5 days post-challenge. Serum was collected on days 20 and 30. (**A**) Daily median rectal temperatures. (**B**) Macroscopic percentage of lung lesions induced from influenza infection with SD435 (H3N2) on 5 d.p.i. (**C**) Macroscopic percentage of lung lesions induced from influenza infection with SD467 (H1N2) on 5 d.p.i. Each bar represents the median value from each group tested. Significant differences are denoted by * (*p* < 0.05), ** (*p* < 0.01). ns = not significant.

We measured the physical response to challenge viruses as well as viral replication in the respiratory tract to assess the protection provided by the bivalent vaccine against these new clinical isolates. Temperature was recorded daily for five days post-viral challenge in all groups. Pigs that were mock vaccinated and challenged with SD435 (H3N2) (MEM/SD435) or SD467 (H1N2) (MEM/SD467) showed a typical temperature spike on day 1 post-viral challenge, with median temperatures of 40.6 °C and 41.1 °C, respectively. This spike was not seen in the vaccinated groups that were challenged with SD435 (Bivalent/SD435) or SD467 (Bivalent/SD467), which had median temperatures of 39.4 °C and 39.6 °C, respectively. On days 2–5 post-challenge, both vaccinated and unvaccinated groups had temperatures around 39 °C (Figure 2A).

Five days post-challenge all pigs were necropsied, with lungs removed in toto and analyzed to quantify the amount of lesions present. The Bivalent/SD435 group showed minimal or no lesions, with a median of 0.65% total lung lesions. The MEM/SD435 group had significantly higher lesions than its vaccinated counterpart, with a median of 5.1% (*p* = 0.0025) (Figure 2B). In the Bivalent/SD467 group, five out of seven pigs had low amounts of lesions (<2%), one had minor lesions (3.75%), and one outlier had high lesions (31%), with a group median of 1.9%. Compared with the vaccinated group, the MEM/SD467 group had a higher degree of lesions with a median of 4.55% (*p* = 0.0417) (Figure 1C).

**Figure 3:**
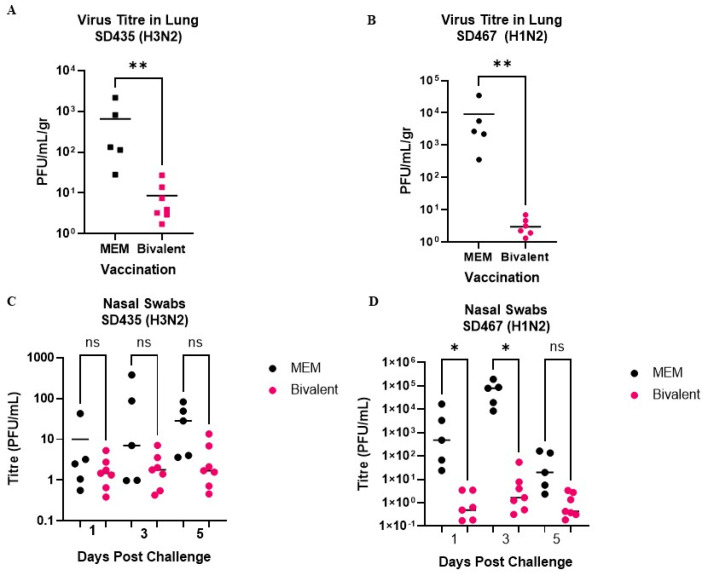
Viral load in the lungs and nasal swabs of pigs infected with SD435 and SD467. Pigs were vaccinated twice with a bivalent vaccine composed of SD191−R342V (1 × 10^6^ PFU) and SD69-K345V (1 × 10^6^ PFU) on days 0 and 21. On day 31, the pigs were intratracheally challenged with 1 × 10^6^ PFU of either SD435 or SD467 and then monitored daily for 5 days post-challenge. Viral titration from the lungs of pigs infected with (**A**) SD435 (H3N2) and (**B**) SD467 (H1N2) as well as in nasal swabs from pigs infected with (**C**) SD435 (H3N2) and (**D**) SD467 (H1N2). Each bar represents the average value from each group tested. Significant differences are denoted by * (*p* < 0.05), ** (*p* < 0.01). ns = not significant.

In the lungs, the Bivalent/SD435 and Bivalent/SD467 groups both had low titers of virus, with averages of 8.6 PFU/mL/gr and 3.0 PFU/mL/gr, respectively. Conversely, MEM/SD435 and MEM/SD467 groups had higher amounts of virus, with averages of 656.1 and 9118.2 PFU/mL/gr, respectively (*p* = 0.0025 for both) (Figure 3A,B). Similar trends were seen in the nasal swabs. In the Bivalent/SD435 group, nasal titers were low on days 1, 3, and 5 post-challenge (dpc), whereas in the MEM/SD435 group titers were slightly elevated and increased as days progressed (ns) (Figure 3C). In the Bivalent/SD467 group, nasal titers were also low, averaging below 5 PFU/mL on days 1 and 5, and 10.0 PFU/mL on day 3 post-challenge. Titers were higher in the MEM/SD467 group on each day, averaging 4123.6 PFU/mL/gr on 1dpc (*p* = 0.0278), 77233.1 PFU/mL/gr (*p* = 0.0009) on 3dpc, and 65.2 PFU/mL/gr on 5dpc (ns) (Figure 3D).

All in all, these results suggest that the bivalent vaccine offered a significant degree of protection against challenge strains, reducing lung lesions and viral replication linked to infection with these two swIAV isolates in the lung and nasal passages.

### 3.2. The Bivalent Vaccine Induces Immune Responses against Challenge Strains

**Figure 4:**
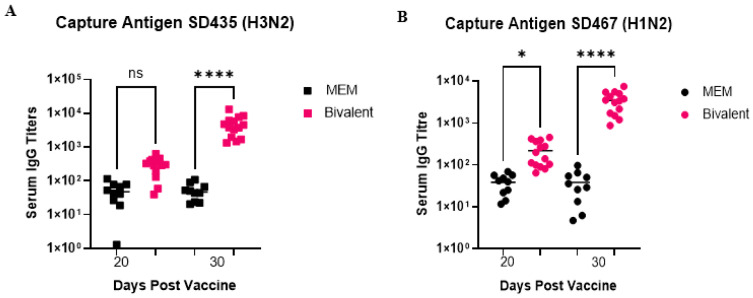
Serum swIAV-specific antibodies induced after bivalent vaccination in pigs. Pigs were vaccinated twice with a bivalent vaccine composed of SD191-R342 V (1 × 10^6^ PFU) and SD69-K345 V (1 × 10^6^ PFU) on days 0 and 21. On day 31, the pigs were intratracheally challenged with 1 × 10^6^ PFU of either SD435 or SD467 and then monitored daily for 5 days post-challenge. Serum was collected on days 20 and 30. Serum IgG levels raised against (**A**) SD435, (**B**) SD467. Each sample was conducted in duplicate. Each bar represents the average value from each group tested. Significant differences are denoted by * (*p* < 0.05), or **** (*p* < 0.0001). ns = not significant.

We measured the antibody response in the serum and lung specific to both challenge strains after prime-boost vaccination with the bivalent vaccine. Serum was collected from pigs after the first vaccine (day 20) and after the second vaccine (day 30). Challenge viruses SD435 (H3N2) and SD467 (H1N2) were used as capture antigens in order to measure the virus-specific IgG antibody response in the serum. With SD435, there was no significant difference between antibody titers in the MEM and the bivalent vaccinated groups after the first vaccination (day 20). However, against SD467 antibody titers were significantly higher in the vaccinated group on day 20 (*p* = 0.0321). After the second vaccine (day 31), antibody titers were significantly higher in the vaccinated group against both SD435 and SD467 than in the MEM mock vaccine groups (*p* < 0.0001) (Figure 4A,B). Specifically, against capture antigen SD435, serum IgG titers in the MEM mock vaccinated group averaged at 52 on days 20 and 30, whereas they were 311 (day 20) and 4852 (day 30) in the bivalent vaccine group (Figure 3A). Against SD467, serum IgG titers in the mock vaccinated MEM group were 39 (day 20) and 38 (day 30), while in the bivalent vaccine group they were 219 (day 20) and 3509 (day 30) (Figure 3B).

**Figure 5:**
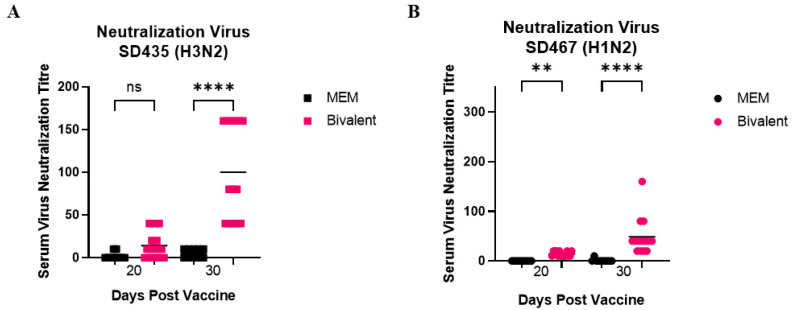
Neutralizing antibodies induced after bivalent vaccination in pigs measured in the serum. Pigs were vaccinated twice with a bivalent vaccine composed of SD191-R342 V (1 × 10^6^ PFU) and SD69-K345 V (1 × 10^6^ PFU) on days 0 and 21. On day 31, the pigs were intratracheally challenged with 1 × 10^6^ PFU of either SD435 or SD467 and then monitored daily for 5 days post-challenge. Serum was collected on days 20 and 30. (**A**) Neutralizing antibody levels in the serum against SD435 (H3N2) (**B**) Neutralizing antibody levels in the serum against SD467 (H1N2). Each sample was conducted in quadruplicate. Each bar represents the average value from each group tested. Significant differences are denoted by ** (*p* < 0.01), or **** (*p* < 0.0001). ns = not significant.

Similar trends were seen when neutralizing antibody titers were measured in the serum against the two challenge strains. Again, there was no significant difference between the neutralizing antibody titers in the MEM and bivalent vaccinated groups against SD435 after one dose of vaccine (day 20) (Figure 5A,B). Against SD467, antibody levels were significantly higher on day 20, after one vaccine (*p* = 0.0069). Against both viruses, there was an increase in antibody titers in the bivalent vaccine groups after the second dose (day 30) (*p* < 0.0001). Titers in the MEM mock-vaccinated group challenged with SD435 averaged 1 (day 20) and 3 (day 30), while titers in the bivalent group averaged at 10 (day 20) and 77 (day 30) (Figure 5A). Titers in the MEM mock-vaccinated group challenged with SD467 averaged at 0 (day 20) and 2 (day 30), while titers in the bivalent vaccine group averaged at 10 (day 20) and 54 (day 30) (Figure 5B).

**Figure 6:**
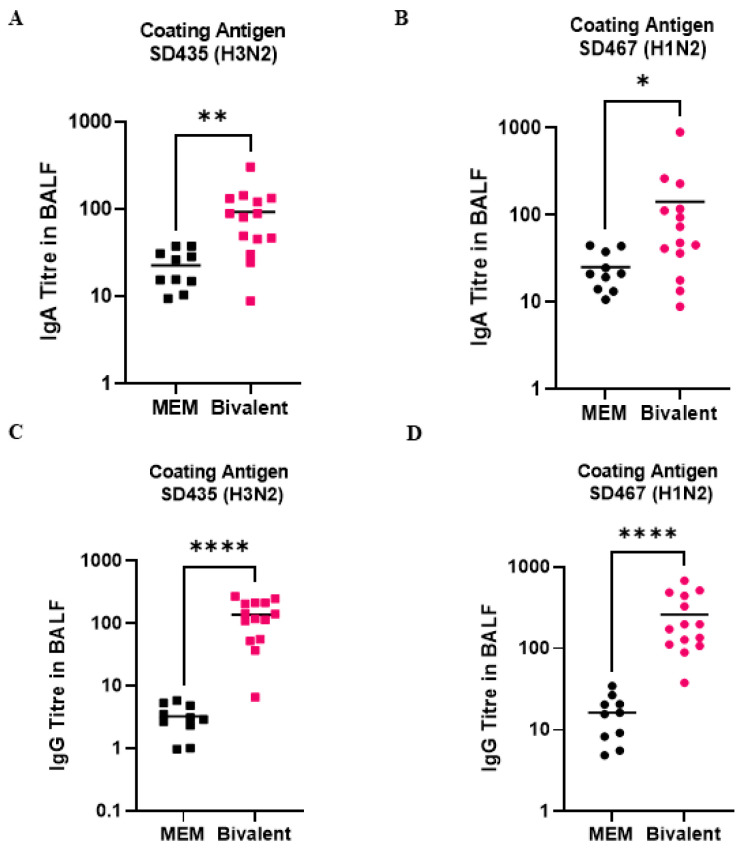
swIAV-specific antibodies induced after bivalent vaccination in pigs measured in the BALF. Pigs were vaccinated twice with a bivalent vaccine composed of SD191-R342 V (1 × 10^6^ PFU) and SD69-K345 V (1 × 10^6^ PFU) on days 0 and 21. On day 31, the pigs were intratracheally challenged with 1 × 10^6^ PFU of either SD435 or SD467 and then monitored daily for 5 days post-challenge. BALF was collected on day 36 at necropsy. (**A**) IgA levels in BALF against SD435 (H3N2), (**B**) IgA levels in BALF against SD467 (H1N2), (**C**) IgG levels in BALF against SD435 (H3N2), and (**D**) IgG levels in BALF against SD467 (H1N2). Each sample was conducted in duplicate. Each bar represents the average value from each group tested. Significant differences are denoted by * (*p* < 0.05), ** (*p* < 0.01), or **** (*p* < 0.0001). ns = not significant.

Upon necropsy (day 36), BALF was collected from each of the pigs so that antibody levels could be measured in the lungs. Challenge viruses SD435 and SD467 were used as capture antigens to measure the virus-specific IgA and IgG response. Against SD435, IgA levels in the MEM mock vaccine groups averaged at 17, whereas titers in the bivalent vaccine group were significantly higher, at an average of 95 (*p* = 0.0014) (Figure 6A). For SD467, IgA levels averaged at 18 in the mock vaccine group and were significantly higher at 158 in the bivalent vaccine group (*p* = 0.0185) (Figure 6B). In terms of IgG, titers against SD435 in the MEM mock vaccine group averaged at 3, while in the bivalent group they were significantly higher at an average of 138 (*p* < 0.0001) (Figure 6C). IgG antibodies against SD467 averaged at 16 in the MEM mock vaccine groups, whereas in the bivalent vaccine group they were significantly higher, at an average of 259 (*p* < 0.0001) (Figure 6D).

**Figure 7:**
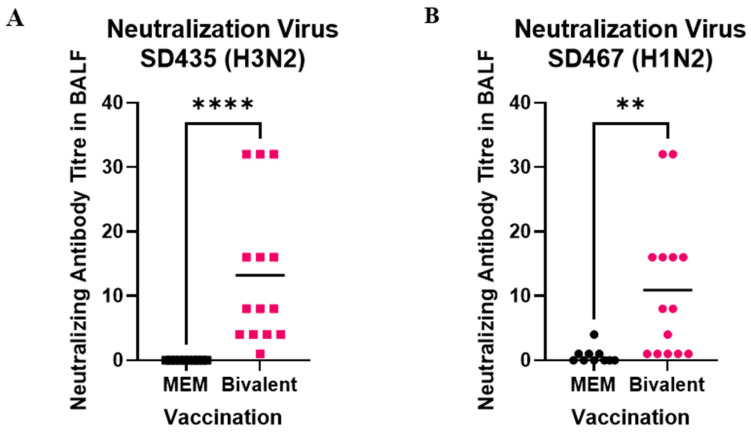
Neutralizing antibodies induced after bivalent vaccination in pigs measured in the BALF. Pigs were vaccinated twice with a bivalent vaccine composed of SD191-R342 V (1 × 10^6^ PFU) and SD69-K345 V (1 × 10^6^ PFU) on days 0 and 21. On day 31, the pigs were intratracheally challenged with 1 × 10^6^ PFU of either SD435 or SD467 and then monitored daily for 5 days post-challenge. BALF was collected on day 36 at necropsy. (**A**) Neutralizing antibody levels in BALF against SD435 (H3N2), (**B**) Neutralizing antibody levels in BALF against SD467 (H1N2). Each sample was conducted in quadruplicate. Each bar represents the average value from each group tested. Significant differences are denoted by ** (*p* < 0.01), or **** (*p* < 0.0001). ns = not significant.

With regards to neutralizing antibodies in the BALF, the trends were similar to those seen in the IgA and IgG ELISAs. Against SD435, neutralizing antibody titers were undetectable in the MEM mock vaccine groups, and they averaged significantly higher at 13.2 in the bivalent vaccine group (*p* < 0.0001) (Figure 7A). Similarly, antibody titers specific to SD467 averaged at 0.7 in the MEM mock vaccine groups, and were significantly higher in the bivalent vaccine group with an average titer of 10.9 (*p* = 0.0002) (Figure 7B). Altogether, this data shows that two doses of the bivalent vaccine induce a potent systemic humoral response, as well as a local immune response in the lung against these two non-homologous clinical isolates.

## 4. Discussion

We previously demonstrated that elastase-dependent viruses SD191−R342V and SD69-K345V were completely attenuated and non-virulent in pigs, and that two vaccinations with this bivalent LAIV elicited a robust immune response and provided protection against infection with homologous SD191 (H1N2) and SD69 (H3N2) strains [14]. In this current study, we wanted to test whether the bivalent vaccine would hold up in vivo against more recent clinical isolates that have undergone antigenic drift. SD467, like SD191, is a member of the Hα-3 antigenic group that has emerged in Canada, but it has acquired numerous mutations in key antigenic sites [12,15]. Likewise, SD435 represents the H3N2 IV-E cluster that is present in western Canada and possesses multiple amino acid substitutions in key H3 antigenic sites from those present in SD69 [17].

The bivalent LAIV significantly reduced lesions in vaccinated pigs when challenged with either SD435 (H3N2) or SD467 (H1N2) and prevented a spike in temperature that was seen in MEM (mock)-vaccinated groups one day post-challenge. It also led to a reduction of viral replication of both strains in the lung, and a reduction of SD467 (H1N2) in the nasal swabs. Interestingly, nasal titers of SD435 (H3N2) were low in both vaccinated and unvaccinated groups, despite identical sampling methods, suggesting this strain may not have as much tropism for the nasal passages. With regards to the outlier pig in Group D, which had a high lung lesion score of 31, temperature measurements showed no spike at challenge, and virus titers in the lung were below 10 PFU/g/mL. Antibody levels in the serum and the local lung response was also the same as all other vaccinated pigs. This leads us to speculate the lesions were not influenza related. Seroanalysis revealed that a strong immune response was mounted against both strains after two doses of the vaccine, and the same proved true with regards to local analysis in the lung. Antibodies directed at surface glycoproteins are paramount in protecting against IAV infection, so the high levels of neutralizing antibodies as well as IgG and IgA found in vaccinated pigs support the protection seen in vivo [20].

Whole inactivated virus (WIV) vaccines are the most commonly available for swine. Traditionally formulated with adjuvant, they are considered a safe approach since there is no risk of reassortment with circulating strains. However, they provide limited efficacy against mismatched strains, and have been shown to lead to vaccine-associated enhanced respiratory disorder (VAERD) when used against mismatched strains. Their efficacy also diminishes in the presence of maternally derived antibodies (MDAs) [4]. Those commercially available in North America include FluSure XP^®^, which is available as a tetravalent formulation in the USA with H1N1, H1N2, and H3N2 clusters IV-A and IV-B [21]. An older formulation of Flusure XP^®^ is available in Canada with two strains of H1N1 and one H1N2 strain, isolated between 2000 and 2005 [22]. In both North American countries, FluSure^®^ Pandemic is available, a monovalent vaccine composed of the H1N1pdm09 strain, as well as Pneumostar SIV Complete (Elanco, Greensboro, North Carolina, US Inc.), which contains H1N1, H1N2, and H3N2, and Pneumostar SIV, with H1N1 and H3N2 subtype strains (GOC, USDA) [23,24]. These commercially available vaccines account for around 50% of swine influenza vaccines in North America, and the other 50% of vaccinations are autogenous vaccines [4].

In terms of alternative vaccine platforms, a recombinant alphavirus-derived replicon particle vaccine was licensed in the USA [4]. This vaccine platform employs an alphavirus with an altered genome, where viral structural genes are replaced by a gene of choice, rendering the alphavirus replication defective. This RNA is self-replicating, so the vaccine platform leads to high expression of the gene of interest, and for influenza both HA and nucleoprotein (NP) have been tested as antigens [25]. Studies have shown that use of this platform provides protection against antigenically HA-matched and -mismatched challenge, as well as NP-mismatched strains, although the platform was not able to provide protection in the presence of MDAs.

The first LAIV for swine influenza was approved by the U.S. Department of Agriculture (USDA) in 2017. Ingelvac Provenza™ is a bivalent H3N2 and H1N1 vaccine, with the HA and NA from two strains isolated in the USA expressed on the TX98 backbone, attenuated through truncation of the nonstructural protein (NS1) [14,26]. LAIVs mimic natural infection and lead to increased mucosal immunity in the upper airways when delivered intranasally. Where inactivated vaccines mainly lead to production of systemic IgG antibodies, live attenuated vaccines can induce mucosal IgA in the respiratory tract, as well as increased cell-mediated response due to the exposure of the immune system to internal influenza proteins, which contain more T cell epitopes [27]. This leads to better protection against mismatched strains. They have shown partial protection in the presence of MDAs. While IgG antibodies are more prevalent in the lower respiratory tract, polymeric IgA antibodies are predominant in the upper respiratory tract of pigs, most often as dimers [28]. These antibodies are produced locally and are transported across the epithelial cell layer where they remain in the mucous, aided by a secretory component that resists degradation by proteases [28,29]. IgA antibodies are the adaptive immune system’s first line of defense against incoming pathogens, working to block viral attachment to sialic acid receptors [30]. Polymeric IgA antibodies have been shown to be more broadly cross-reactive than monomeric IgG antibodies, potentially due to multivalent binding [31]. Studies have also shown that these antibodies can prevent release of newly formed IAV from infected cells much more efficiently than IgG or monomeric IgA, which can be found in porcine serum, suggesting the polymeric structure of IgA is advantageous for cross-linking the viral progeny to HA expressed on the infected cell surface [31,32,33]. The local IgA antibody response is therefore integral in the protection against infection with IAV and has been suggested to be a correlate of protection in humans [34].

However, the risk with LAIV is the potential for reassortment with circulating strains. A phylogenetic study in the USA found new strains in circulation that had reassorted with the vaccine strains included in Ingelvac Provenza™ [26]. The elastase-dependent LAIV platform reduces this risk, as the elastase protein is very scarce in the porcine respiratory tract, so replication of vaccine viruses is very restricted, as is the time frame for reassortment to occur. Future studies will include the evaluation of this bivalent vaccine’s reassortment risk, as well as how this vaccine holds up in the presence of MDAs. It would also be interesting to test the cell-mediated response of this vaccine, as this is one of the major benefits of LAIV. In conclusion, the bivalent elastase-dependent LAIV extended protection to new clinical isolates found in western Canada, and would fill some gaps in the swine influenza vaccine market.

## Data Availability

The data and analyses of this study are all reported in this article.
